# An Analysis of Epidemiological Factors in Heart Failure Outcomes

**DOI:** 10.7759/cureus.22627

**Published:** 2022-02-26

**Authors:** Karan Patel, Kamil Taneja, Aleem Mohamed, Sai Batchu, Hailey Hsiung, Connor Mott, Haley Tornberg, Urvish K Patel

**Affiliations:** 1 Medical Student, Cooper Medical School of Rowan University, Camden, USA; 2 Medical Student, Stony Brook University, Stony Brook, USA; 3 Neurosciences, Independent Researcher, Montville, USA; 4 Healthcare (Social Sciences), Independent, Highland Park, USA; 5 Public Health and Neurology, Icahn School of Medicine at Mount Sinai, New York, USA

**Keywords:** healthcare disparity, length of hospital stay (los), insurance status, epidemiology and biostatistics, heart failure

## Abstract

Background

Various socioeconomic and demographic factors play a role in determining treatment outcomes across numerous conditions. Different studies have shown that certain demographic factors, such as income status, directly correlate with treatment outcomes. In this study, we analyze the effect of some of these variables, namely, insurance and age, on various endpoints, including length of stay and discharge status, among heart failure patients.

Methodology

The data used in this project were retrieved from the HealthCare Utilization Project. We sorted the data by insurance, age, length of stay, and discharge status. To compare discharge status between different insurance types and age groups, we used Stata to compute odds ratios and 95% confidence intervals. To compare the length of stay among different age groups and insurance types, we conducted an unpaired two-tailed Student’s t-test.

Results

Across all age groups, we found that younger patients with heart failure are more likely to discharge against medical advice compared to older patients. The average length of stay for heart failure patients was the same across all age groups except those 85 and older. Moreover, patients with a lower socioeconomic status, as determined by insurance type, were more likely to discharge against medical advice and less likely to die within hospitals.

Conclusions

Our results speak to the socioeconomic inequalities seen in medicine today. Studies have shown that those with a lower socioeconomic status tend to have worse outcomes across various conditions. Our analysis shows this phenomenon holds true for heart failure as well. In addition, our study helps to determine which groups are at higher risk of making medical decisions, such as discharging against medical advice, that will negatively affect their condition. Identifying these high-risk groups is a key first step to counteracting such behavior.

## Introduction

Heart disease is the leading cause of death in the United States, accounting for one in every four deaths [1,2]. Although there are many different diseases that fall under the umbrella term of heart disease, in this article, we focus on heart failure, the decreased ability of the heart to pump sufficient blood to the body. Within this subset of heart disease, heart failure is responsible for an estimated $39.2 billion costs annually and affects roughly 23 million individuals worldwide across all age groups [3,4]. In addition to these effects, heart failure was delineated as a growing epidemic in 1997, making this disease an important topic to investigate further.

The pathophysiology, diagnostic classifications, and treatment algorithms of heart failure have been well-described and identified [5-7]. However, there is a lack of studies analyzing the impact of certain demographic factors in heart failure patients within the context of a patient discharging against medical advice (AMA). Demographic factors such as gender, age, race, and insurance status are contributing factors under the comprehensive topic of healthcare disparities, a growing concern in the field of medicine that has significantly affected patient care and outcomes. Specifically, insurance status has been previously linked to mortality with a potential 5-25% decrease in mortality in insured patients compared to their uninsured counterparts [7]. Additionally, lower socioeconomic status, a proxy term for insurance status, has been shown to contribute to patients receiving poor prognoses and experiencing worse outcomes across a range of different conditions [8-10].

On the topic of patients with heart failure being discharged, there are two avenues: per doctor’s orders or AMA. Patients who are discharged AMA have been shown to have significantly higher readmission rates and are more likely to experience severe disease manifestations [11-13]. For example, a study conducted by Tan et al. found a 9% increase in readmission rate for those who were discharged AMA compared to those who did not discharge AMA. Hence, determining which populations are more prone to be discharged AMA can be a key first step in addressing this phenomenon [11].

In this study, we aimed to examine the effect of age and insurance status on the length of stay, death rate, and discharge AMA within the patient population affected by heart failure.

## Materials and methods

We conducted a retrospective analysis with data retrieved from the Health Cost and Utilization Project website. Within this database, we used the following inputs to generate a dataset: setting of care: inpatient data only; diagnosis: clinical classification software redefined (CCSR); heart failure, outcomes, and measures: length of stay, discharge, and deaths; geographic setting: national; patient characteristics: age groups and payer status; and year: 2018. After generating this dataset, we then ran six separate sets of analysis. To analyze the mean length of hospital stay between different age groups and among those with different types of insurance, we use an unpaired two-tailed Student’s t-test. Afterward, we performed a separate analysis looking at the rates of discharge against/with medical advice and death/discharge among various age groups and insurance types. For these analyses, we used Stata (StataCorp LP, College Station, TX, USA) to perform odds ratios along with 95% confidence intervals.

## Results

**Figure 1:**
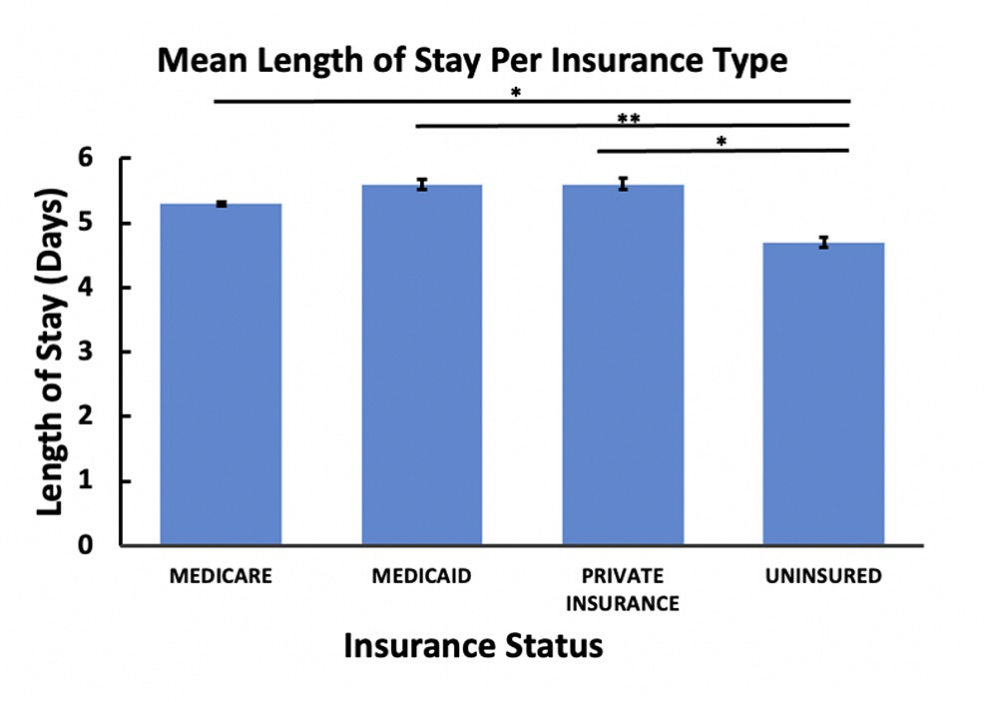
Average length of hospital stay by insurance type. *: P < 0.05; **: P < 0.01

**Figure 2:**
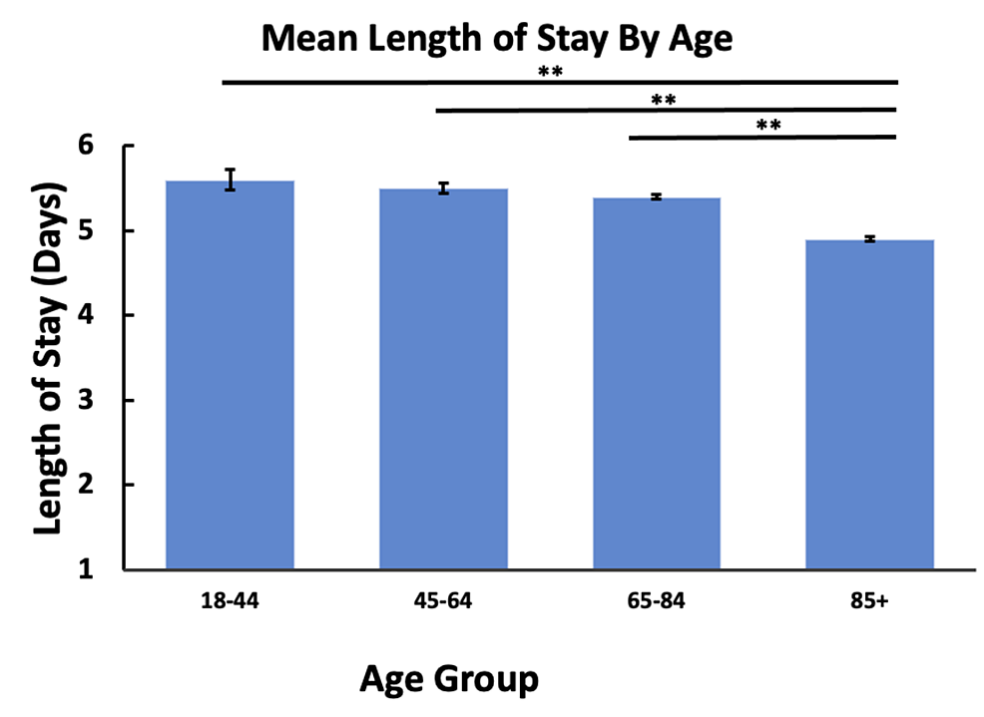
Average length of hospital stay by age. **: P < 0.01

Our results show that across all age groups, younger patients were more likely to discharge AMA (Table 1). In conjunction with this, younger patients were also more likely to die in hospitals compared to older patients, as shown in Table 1. We found that socioeconomic status, as determined by insurance type, played a role in both the likelihood of someone discharging AMA and in the average length of stay (Table 1, Figure 1). Patients with a lower socioeconomic status, as determined by insurance type, were more likely to discharge AMA and had shorter hospital stays (Table 2, Figure 1). Furthermore, a patient’s age did not appear to play a major role in the mean length of stay. We found no significant differences in the average length of stay across all age groups besides those who were older than 85 years; these patients were more likely to have a shorter length of stay compared to all age groups (Figure 2).

**Table 1:** Odds ratios: discharge against medical advice versus discharge with medical advice. CI: confidence interval

	Odds ratios (95% CI)
Age group (years)	
85+	Reference
18–44	17.83 (16.41-19.83)
45–64	12.41 (11.51-13.38)
65–84	3.25 (3.01-3.52)
Insurance type	
Uninsured	Reference
Medicare	0.20 (0.19-0.21)
Medicaid	1.15 (1.09-1.21)
Private insurance	0.28 (0.26-0.30)

**Table 2:** Odds ratios: death in hospital versus discharge. CI: confidence interval

	Odds ratios (95% CI)
Age group (years)	
18–44	Reference
45–64	0.96 (0.88-1.04)
65–84	0.49 (0.45-0.53)
85+	0.31 (0.29-0.34)
Insurance type	
Uninsured	Reference
Medicare	2.13 (1.94-2.33)
Medicaid	1.05 (0.94-1.16)
Private insurance	1.88 (1.70-2.07)

## Discussion

Based on our analyses, we found that younger patients were significantly more likely to discharge against doctors’ orders compared to older patients. This held true for all age group comparisons for younger versus older patients. While the exact reasons for this are unclear, we suspect they are multifactorial in nature. Younger patients tend to report poorer experiences in inpatient settings compared to older patients, which may make them more prone to leaving these settings earlier [14]. In addition, it has been shown that older patients are at a higher risk of experiencing adverse effects of heart failure treatment. For example, they are more likely to experience dehydration and electrolyte imbalances with the use of diuretics compared to their younger counterparts [15]. Adding in the factor that older patients often have multiple comorbidities and experience polypharmacy further complicates the diagnosis, treatment, and management of heart failure [16-18]. As a result, they may be less inclined to leave the hospital setting against physicians’ orders because they generally take longer to recover from treatment and may potentially require a longer treatment period.

One anomaly that our analysis uncovered was that patients aged 85 and older were actually more likely to have shorter hospital stays than all other age groups. While this can be attributed to the aforementioned statistic of older patients having a significantly higher death rate due to HF, we also note that the factor of the length of hospital stays does not take into account the frequency of visits for heart failure [19]. We hypothesize that older patients, while having shorter stays, may visit the hospital more often due to HF; however, this was not a factor that we could control for in this study. Overall, the trends for age uncovered by our analysis fall in the line with the general body of literature on patient psychology and mortality [20].

The trends that we discovered for insurance also fall in line with general expectations. According to our analysis, uninsured patients had shorter hospital stays compared to patients on all other forms of insurance. Additionally, uninsured patients and those on Medicaid were more likely to sign out AMA compared to patients on private insurance and Medicare. Other studies analyzing different diseases have found similar trends of shorter hospital stays among uninsured patients [19-21]. While this finding makes sense from a monetary perspective, it is also paradoxical because uninsured patients are twice as likely to report going without needed care. As a result, they should have more severe disease manifestations when presenting to the hospital [22]. However, having to burden out-of-pocket costs may be one driving factor leading to shorter hospital stays. In addition, hospitals are less inclined to prolong care for uninsured patients. One study found that uninsured patients were 66% more likely to be discharged from hospitals compared to those on private insurance [23]. The results of our study further speak to the inequalities present in healthcare today.

In our analysis, we found that patients on Medicaid and uninsured patients were less likely to die in hospitals because of heart failure, while Medicare patients were most likely to die in hospitals because of heart failure. While this may seem paradoxical, it fits in with the general trend of socioeconomic inequalities seen in healthcare. As found in our analysis, patients with Medicaid and no insurance are more likely to sign out AMA which may contribute to lower hospital death rates. In addition, because of data unavailability, our analysis did not look at out-of-hospital death rates for these groups. Patients with these insurance types may be less likely to get healthcare/get inferior healthcare in general and, as a result, do not contribute to the “in-hospital” death rate statistic [24-26]. Therefore our expectation is that the community death rate for patients on these insurance types with a heart failure diagnosis would be significantly higher than those with Medicare and private insurance. Conversely, Medicare patients generally tend to be 65+ (outside of those with disabilities), placing them in one of the two groups (65-84 and 85+) least likely to check out AMA. In addition, Medicare is not income-based and so these patients are not as motivated as those with a lower socioeconomic status to leave a healthcare setting.

Some limitations in this study were that we could not adjust for the severity of heart failure and comorbidities among patients. General trends have shown that patients with lower socioeconomic status and older-age patients often have multiple comorbidities which may affect their length of stay. However, the general trends that we see in our study have also been shown to be true in other studies analyzing different medical conditions [27].

## Conclusions

Our study supported two separate notions: (1) younger patients are more likely to discharge AMA, and (2) insurance status plays a significant role both in the likelihood that a patient discharges AMA and their length of stay.

While the relationship between patient age and rate of discharge against physician advice is not surprising, it is clinically important. Any patient leaving AMA could experience unforeseen consequences which can result in repeated hospitalizations, increasing both physical and financial burdens on the patient. Providing this at-risk population with adequate treatment has a tremendous impact on their overall quality of life.

The socioeconomic status of a patient is a defined social determinant of health, and our analysis supports the notion of a continued dissonance between insurance status and patient care. There is a multitude of reasons for how insurance status can affect a patient’s care and length of stay, some of which may be concerns of costs of hospital stay and transportation to the hospital; however, more research is needed to delineate these factors fully as they are quite diverse and varying in nature.
